# Generation of rule-based matrices with the matRiks package: a tutorial

**DOI:** 10.3389/fpsyg.2025.1698995

**Published:** 2026-01-28

**Authors:** Ottavia M. Epifania, Andrea Brancaccio, Pasquale Anselmi, Debora de Chiusole

**Affiliations:** 1 Department of Psychology and Cognitive Science, University of Trento, Rovereto, Italy; 2 Psicostat Research Group, University of Padova, Padova, Italy; 3 Department of Philosophy, Sociology, Education and Applied Psychology, University of Padova, Padova, Italy; 4 Institute for Applied Mathematics and Information Technologies “E. Magenes”, National Research Council, Milan, Italy

**Keywords:** fluid intelligence, open-source, R package, reproducibility, rule-based matrices

## Abstract

Few resources are available for the automatic generation of Raven-like matrices. Some of them are no longer working, while others are hardly customizable without advanced programming skills. Although an R package exists for generating stimuli for psychological assessments, it currently only supports creating rotations of the same shape. The matRiks package has been developed to address the above-mentioned issues. This package generates matrices based on different types of transformation rules. Some rely on visuospatial features, such as shape, size, or orientation. Others are based on logical operations, such as set intersection or union. The package also introduces a key innovation, the automatic generation of distractors based on the common error patterns observed in Raven's tests. This allows for the construction of realistic and diagnostically meaningful response options for each matrix. Overall, the matRiks package provides a flexible and systematic framework for generating well-structured and cognitively interpretable matrix reasoning tasks, based on clearly defined transformation rules. It enables precise control over matrix complexity and supports scalable matrix creation for experimental and assessment purposes. Developed in the R environment, the matRiks package is fully open-source, facilitates the reproducibility of stimuli, and is designed to be easy to use for people with basic knowledge of the R language.

## 1 Introduction

Cattell (1963) defined fluid intelligence (FI) as the ability to solve novel reasoning problems that have little to do with concepts learned in schools or through acculturation processes. The term “fluid” highlights the flexibility of this form of intelligence, which can be applied across a wide range of tasks and cognitive domains (Horn, 1972). Given this definition of FI, it appears natural that the instruments used for its evaluation are designed on the respondent's ability to solve abstract problems that involve acculturation as little as possible, such as figurative analogies, figure classifications, matrices, and number and letter series (Horn, 1968).

The Raven's Progressive Matrices (RPM; Raven, 1938, 1962) are among the most widely known tools for assessing FI. The RPM consists of a series of non-verbal, multiple-choice items in which respondents are asked to complete visual patterns, composed of abstract drawings, by identifying the underlying features and rules that govern the relationships among the elements. These visual patterns are commonly referred to as *matrices*, which can be composed of either four cells (denoted as 2 × 2 matrices) or nine cells (denoted as 3 × 3 matrices). To solve each matrix correctly, respondents must select, from a set of alternative options, the figure that best completes the pattern. The task is designed to assess the individual's ability to detect and apply the abstract rules that structure the relationships among the figures manipulated in the matrix. Different versions of the RPM have been developed to accommodate different target populations in terms of age and cognitive ability. The Standard Progressive Matrices (SPM; Raven, 1938) are designed for the general population and consist primarily of 3 × 3 matrices with eight response options, arranged in five sets (A–E) of increasing difficulty. The Colored Progressive Matrices (CPM; Raven, 1962) are intended for children under 12 years of age, older adults, and individuals with cognitive or learning difficulties. This version includes colored stimuli in the first two sets (A and Ab), followed by a black-and-white set (B). The matrices are primarily in a 2 × 2 format with six response options, focusing on lower levels of difficulty to enhance accessibility. Additionally, the CPM includes “puzzle” items in which a piece is missing from a global configuration rather than from a matrix structure. In contrast, the Advanced Progressive Matrices (APM; Raven et al., 1977) are designed for individuals with above-average intellectual ability, such as university students or gifted populations. After a brief practice set, the APM presents a single set of 3 × 3 black-and-white matrices with eight response options and substantially higher complexity, designed to assess higher-order abstract reasoning and to discriminate performance at the upper end of the ability distribution. Unlike the SPM and CPM, which are typically scored based on accuracy alone (i.e., the number of correct responses), the APM often considers both accuracy and response time as performance indices.

The RPM and similar tasks (here denoted as Raven-like matrices) are employed in different fields, from clinical evaluation of FI to the selection processes in organizational psychology (Payne et al., 2007; Potosky et al., 2005; Salgado et al., 2003; Schmidt et al., 1986; Van Iddekinge et al., 2012). Since Raven-like tasks are designed to assess the ability to solve novel and abstract problems, it is essential to preserve the novelty of the stimuli by avoiding widespread dissemination. However, creating such stimuli is a complex and time-consuming process that requires careful design to ensure appropriate difficulty levels and rule structures. To pursue this aim, researchers in the field have increasingly explored automated methods for generating matrices to produce large sets of novel matrices while reducing the effort required for manual construction and providing a useful means to calibrate their complexity.

One effective approach to automated matrix generation leverages the systematic combination of transformation rules, enabling the creation of a wide variety of novel stimuli while maintaining control over their structural properties. By combining the rules underlying matrix generation, researchers can produce matrices with varying levels of complexity, ensuring both novelty and gradual progression in challenge. Over the years, several tools have been developed to support this goal (Blum and Holling, 2018; Matzen et al., 2010; Thimbleby, 2018; Wang and Su, 2015). Although these tools have played an important role in laying the groundwork for automated matrix generation, each presents limitations that constrain their broader applicability, such as a lack of code maintenance, limited flexibility without programming knowledge, or limited rule diversity. For further details on these methods and their specific features, the reader is referred to Section 2.

To address these issues, a new R (R Core Team, 2025) package, called matRiks (Brancaccio et al., 2023, 2025), has been developed. This tool enables the automatic construction of Raven-like matrices by combining one or more transformation rules applied to one or more geometric figures. In addition to the predefined figures and rules, matRiks allows users to customize the stimuli by introducing new shapes, modifying rule parameters, and adjusting the structure of the response list. Crucially, the systematic manipulation of rules and figures enables researchers to finely control the complexity of the items, either by varying one element at a time or by creating multiple stimuli that share the same underlying rules but differ in surface features. A key innovation of the matRiks package is that it implements the classification of error types known in the literature (see, e.g., Kunda et al., 2016) to generate the response options associated with each matrix. To the best of our knowledge, this feature has not been implemented in the existing tools.

The manuscript is organized as follows. Section 2 provides a comprehensive background on the available methods for constructing Raven-like matrices, detailing existing tools for stimulus generation, the taxonomy of transformation rules, and the classification of distractor types based on cognitive error patterns. Section **??** describes the logic underlying the matRiks package, illustrating how matrices are constructed through the systematic manipulation of figures and rules, and how distractors are consequently generated. The conceptual illustration of the core concepts of the matRiks package is supported by hands-on examples. This is followed by Section 4, which illustrates a guided tutorial for the complete example of stimulus generation, including both the matrix and its associated response list. The manuscript concludes with Section 5 describing some final remarks on the potential applications and future development of the package.

## 2 Automatic generation of Raven-like matrices: State of the art

Many authors have long been engaged in the automatic generation of test items or stimuli, recognizing their potential for improving test efficiency, scalability, and psychometric quality. Pioneering work by Embretson (1999) and Embretson and Reise (2002) introduced cognitively-based item generation, demonstrating how formal models of task performance can guide the systematic production of verbal, mathematical, and spatial items. This line of research emphasized the role of cognitive theories in defining item complexity and dimensionality, thereby laying a solid foundation for subsequent work on automatic item generation (AIG). Subsequent developments have expanded AIG frameworks through item models and cognitive components, as proposed by Gierl and Lai (2012), making these approaches applicable across diverse testing domains.

Despite this growing literature, only a limited number of studies have focused specifically on the automatic generation of matrix-based reasoning tasks, such as those inspired by RPM. Notable examples include the Sandia Matrices (Matzen et al., 2010; Harris et al., 2020), Corvus (Thimbleby, 2018), IMaK (Blum and Holling, 2018), and more recent AI-driven approaches (Wang and Su, 2015; Barrett et al., 2018). These efforts aim to combine rule-based generation with cognitive plausibility, but differ in terms of their theoretical grounding and visual complexity. Psychometric validation of the resulting tests can be performed in subsequent steps using established modeling approaches, such as Item Response Theory (IRT; Matzen et al., 2010) or with a multi-method approach (de Chiusole et al., 2025).

One of the most prominent examples of automatically generated Raven-like stimuli is the Sandia Matrices (Matzen et al., 2010; Harris et al., 2020). The items included in Sandia are produced by a dedicated software developed in Java, which generates matrix reasoning problems based on a set of predefined cognitive transformation rules. Specifically, five types of visual transformations (shape, fill pattern, orientation, size, and number) are systematically combined and applied in multiple directions to create a large variety of unique stimuli. The software offers a limited but versatile set of geometric shapes, including ovals, triangles, rectangles, and trapezoids. Each shape can be manipulated across five gradations for each visual attribute, enabling a combinatorial explosion of possible matrices. Recently, the test construction and analysis of respondent performance were tested by using IRT (Harris et al., 2020). Results support the generation of tests that are both scalable in terms of complexity and psychometrically sound. However, while the systematic application of transformation rules ensures consistency and control, the resulting items can sometimes appear basic, which may limit ecological validity. More importantly, the transformation rules employed at Sandia do not capture the complexity of higher-order human reasoning processes, such as logical operations.

Corvus represents a JavaScript alternative resource for generating Raven-like tasks (Thimbleby, 2018). Its author provided a user-friendly graphical interface that allows the user to specify the figures and the rules for generating the matrices. However, Corvus provides few degrees of freedom in terms of both the figures and the number of rules that can be manipulated through the graphical interface. Any user customization, such as adding other figures, modifying existing ones, or implementing new rules, requires modifying the JavaScript code, which might be quite demanding for users with little experience in JavaScript.

A possible easier alternative might be to use the R language, as the IMaK package does for generating visual analogies (Blum and Holling, 2018). The code for generating such stimuli (along with their response options) is quite straightforward to use. However, the stimuli generated by the package are based solely on the spatial rotation of the figures, with some objects that can be added or removed.

Recent work in artificial intelligence (AI) has tackled both the generation and solution of Raven-like matrix problems. For example, Wang and Su (2015) proposed a symbolic formalization of matrix elements, but their work does not provide a practical way to produce the stimuli. In parallel, AI benchmark datasets (e.g., Synthetic Visual Reasoning Test and Procedurally Generated Matrices; Fleuret et al., 2011; Barrett et al., 2018) enable large-scale evaluation of abstract reasoning in neural networks. Yet, their lack of psychometric validation limits their direct use for assessing human cognitive abilities.

### 2.1 The cognitive task behind Raven-like matrices

As discussed in the previous section, analyzing the cognitive processes individuals engage in when solving Raven-like matrices provides a valuable foundation for the automatic generation of such stimuli. In particular, decomposing the problem-solving task into distinct mental operations enables researchers to formalize the underlying cognitive strategies. These operations can then be translated into well-defined *transformation rules*, which can be systematically implemented in generative algorithms.

Different strategies can be used to solve Raven-like matrices, such as insight-based or elimination-based approaches. Nevertheless, the task that a person is supposed to apply can be described as follows. An individual begins by visualizing the entire matrix. The initial scan helps to recognize recurring visual elements. The individual then attempts to detect systematic variations or relationships across rows and columns, often by comparing adjacent cells. This involves identifying potential transformation rules (e.g., rotation, change in size, or color) that consistently govern how features evolve from one cell to the next. As patterns emerge, the individual formulates hypotheses about the underlying rules and mentally tests them across the matrix to verify their consistency. Finally, the individual applies the inferred rules to the incomplete part of the matrix to select the response option that completes the pattern.

To formalize a similar process, two elements are fundamental: (i) defining the transformation rules that can be manipulated within a matrix, and (ii) designing response options that are cognitively plausible. This implies that the incorrect response options (i.e., distractors) must be created so that they are neither overly obvious nor irrelevant, but instead require the individual to engage in the same mental operations used to solve the matrix. In other words, the distractors should be generated to resemble the structure and surface features of the correct response, thereby ensuring that only the respondents who correctly identify and apply the underlying transformation rules can derive the correct solution. This minimizes the likelihood of solving the matrix through superficial strategies or guessing, thereby preserving the diagnostic value of the task. The following two sections provide a theoretical overview of these two fundamental aspects.

### 2.2 Transformation rules used for the construction of Raven-like matrices

Transformation rules, also referred to as mental operations, problem types, or relations, are the procedures used to construct Raven-like matrices. They represent the generative grammar underlying matrix-based problems, providing the core paradigms for formalizing and structuring the items. By combining these rules, it becomes possible to generate a variety of matrices with diverse patterns and characteristics, thereby creating comprehensive assessments capable of measuring FI across different cognitive domains.

Table 1 presents a systematic classification of the transformation rules currently documented in the scientific literature. This taxonomy was developed to produce a classification that is as objective and generalizable as possible, drawing on contributions from multiple researchers in the field (Harris et al., 2020; Matzen et al., 2010; Deshon and Jepsen, 1995; DeShon et al., 1995). The resulting framework supports the automatic generation of a large and diverse database of Raven-like matrices, varying in structure and complexity, and suitable for assessing FI across a broad age range. Thus, this database serves as the foundation for the AIG system implemented in the matRiks package.

**Table 1:** Generative rules used in matrix construction.

**Rule names**	**Macro-category**	**Description**
Conjunction, “AND”, intersection	Logical	The third cell contains only the shapes present in both the first and second cells (e.g., if cell 1 has shapes A and B, and cell 2 has B and C, cell 3 contains B)
Disjunction, “OR”, superimposition	Logical	The third cell contains the union of all shapes appearing in either the first or second cell of that row or column
Exclusive disjunction, “XOR”, Superimposition with cancellation	Logical	The third cell contains shapes appearing exclusively in the first or second cell, but not in both (i.e., shapes in either cell 1 or cell 2, but not shared).
Superimposition with conditional positioning	Logical	The third cell contains all shapes from the first and second cells, with some elements visually positioned in the foreground and others in the background to indicate layering
Object addition	Logical	The visual combination of objects from the first and second cells into a single merged object, representing the addition of features.
Object subtraction	Logical	The visual removal of the object present in the second cell from the object in the first cell, representing subtraction of features
Mental transformation	Logical	A transformation applied to an object based on a cue provided by a second object. For example, a square in the first cell and a dashed line in the second cell combine to produce a square with dashed sides in the third cell
Quantitative progression, changes in numerosity	Quantitative	A numerical increase or decrease in the number of features across rows or columns (e.g., the number of dots increases progressively from left to right)
Paired quantitative progression	Quantitative	Each cell is divided into two distinct sections (top-bottom or right-left), and quantitative transformations are applied independently to each section
Distribution of two values	Verbal-analytical	Two categorical values are distributed along a row, where the third value is either absent (null) or categorically inconsistent with the other features
Distribution of three values	Verbal-analytical	Three categorical values are distributed along a row or column. For example, each row may contain a triangle, a circle, and a square, each representing a distinct category
Movement, seriality	Visuospatial	The perception of movement is based on a stable background that serves as a reference frame; an object's position changes incrementally frame by frame relative to this background
Rotation, changes in orientation	Visuospatial	The mental or visual rotation of an object to make differently oriented shapes compatible, such as rotating a shape 90 degrees to align it with others
Changes in shape	Visuospatial	A change occurs in the type or form of the shape, for example, from a triangle to a square
Changes in filling	Visuospatial	A change occurs in the shading, texture, or internal pattern of the figure, such as from solid fill to striped or dotted patterns
Changes in size	Visuospatial	A change occurs in the dimensions of the shape: length for open shapes, or area for closed shapes (e.g., the figure becomes larger or smaller)
Changes in edge	Visuospatial	A change involves the perimeter or contour of the figure, such as altering the outline from smooth to jagged

For each transformation rule (rows of the table), Column 1 reports the name, Column 2 indicates the corresponding macro-category, followed by a description (Column 3). It is worth noticing that the classification of the rules into macro-categories is proposed here with two aims: (i) to simplify the exposition and highlight analogies and differences among the rules, and (ii) to streamline their implementations in matRiks package. The considered macro-categories are: (i) *Logical rules*, which involve a logical-deductive process whereby certain premises (i.e., the attributes present in the cells of the matrix) lead to a necessarily consequent conclusion through deduction; (ii) *Visuospatial rules*, which refer to processes related to the encoding and memorization of an object's graphical representation through visual perception in space; (iii) *Verbal-analytical rules*, which require a propositional representation of the stimuli to find the solution to the problem; (iv) *Quantitative rules*, which involve a quantitative changing of the objects.

All the transformation rules can be manipulated according to directional logic. Specifically, the rules can be applied horizontally (i.e., the manipulation of the rule can be seen across the columns of the matrix but not across its rows, H direction), vertically (i.e., the manipulation of the rule can be seen across the rows of the matrix but not across its columns, V direction), or diagonally (i.e., the manipulation of the rule can be seen both across the columns and the rows of the matrix). Regarding the diagonal directional logic, it can follow either the main diagonal of the matrix (i.e., the manipulation of the rule can be seen from the top-left corner to the bottom-right corner, TL-LR direction) or the secondary diagonal of the matrix (i.e., the manipulation of the rule can be seen from the bottom-left corner to the top-right corner, LL-TR direction).

To classify the rules, several relevant articles on the topic were analyzed: Inference rules were extracted from the studies by Harris et al. (2020) and Matzen et al. (2010) on rule-guided automatic stimulus generation; Visuospatial rules referred to the study by Deshon and Jepsen (1995) on Raven's Advanced Progressive Matrices; The study by DeShon et al. (1995) led to the classification of Verbal-analytical rules. Thanks to these previous works, it was possible to build a complete and exhaustive taxonomy of the transformation rules used for constructing the matrices.

While precise difficulty estimates for individual rules or combinations of rules cannot be specified a priori, it is nevertheless possible to advance theory-driven hypotheses about their relative cognitive demands. In particular, developmental theories of cognition, such as Piaget's theory of cognitive development (e.g., Piaget, 1952), can provide a useful conceptual framework. From this perspective, rules involving logical transformations may plausibly engage cognitive processes associated with the formal operational stage and therefore impose higher cognitive demands. In contrast, visuospatial or perceptual transformation rules, which rely more heavily on concrete manipulation or pattern completion, may be more consistent with abilities characteristic of earlier developmental stages and thus plausibly simpler. Although such assumptions cannot substitute for empirical calibration, they may offer informative heuristics for selecting and combining rule types during test design and for generating testable hypotheses about item difficulty across populations. Moreover, evidence from the IRT-based validation of the MatriKS test (see de Chiusole et al., 2025) suggests that matrix difficulty does not necessarily depend on matrix dimensionality *per se* (i.e., 3 × 3 matrices are not inherently more difficult than 2 × 2 matrices). Rather, difficulty appears to be more strongly related to the type of rules involved, the directional logic by which they are manipulated, and, within the framework of the presented package, the number of layers stratified during item construction. For example, holding the number of layers and the set of manipulated rules constant, it is reasonable to expect that logical rule manipulation yields more complex matrices than visuospatial rule manipulation. Similarly, the use of diagonal directional logic may increase item complexity even when only visuospatial rules are involved, particularly when respondents' age is taken into account. Nonetheless, systematic empirical studies are needed to investigate the interactions among the multiple factors that plausibly contribute to matrix complexity, including matrix dimensionality, the number and type of rules, the number of layers, and directional manipulation logic.

### 2.3 The response options

The role of distractors in the response processes involved in solving the Raven matrices has been investigated mostly by examining the specific error responses chosen by respondents (see, e.g., Kunda et al., 2016; Storme et al., 2019). The underlying logic is that the incorrect response is not chosen at random by the respondent, but may result from an educated guess or from being misled by a specific feature. In other words, the incorrect responses might reflect an inappropriate solution strategy, leading to the choice of one type of distractor over another (Kunda et al., 2016). The distractors can be classified according to the incorrect response strategy that they represent. Kunda et al. (2016) presents a list of criteria for the identification of the distractors in the SPM based on the classification of the error types from the manuals of the Colored Progressive Matrices and of the Advanced Progressive Matrices (Raven and Raven, 2004). The distractor that is chosen in place of the correct response option can be collected into four main conceptual errors: (i) Repetition (R) errors occur when the chosen response option is a cell adjacent to the blank cell, (ii) Difference (D) errors occur when the chosen response option is completely different from any entry of the matrix, (iii) Wrong Principle (WP) errors occur when the chosen response option follows rules other than the ones used in the matrix, and (iv) Incomplete Correlate (IC) errors occur when the chosen response option is in fact the correct response with a variation on a single feature.

The characteristics of the specific conceptual errors can be further categorized within the main categories. For example, errors falling under the R category can be further classified by their position: to the left of the blank cell (R-Left), on top of it (R-Top), or on the top-left of the blank cell (R-Diagonal). The specific errors in the WP category might involve repeating a cell that is not adjacent to a blank space (WP-Copy) or combining elements from different cells in a way that does not follow the rules used to create the matrix (WP-Matrix). Some specific errors in the IC category include changes to the orientation of the correct response (i.e., IC-Flip), changes in the color of the correct response (i.e., IC-Neg), changes in the size of the correct response (i.e., IC-Size), or the omission of an element from the correct response (i.e., IC-Inc). Finally, regarding the D category, specific errors might include a cell being completely white or black (D-Blank) or the merging of different cells within the matrix (D-Union). The interested reader can find an exhaustive discussion on the error types in Kunda et al. (2016).

## 3 The matRiks package

The matRiks package (Brancaccio et al., 2023, 2025) generates 2 × 2 and 3 × 3 Raven-like matrices with their corresponding set of responses (i.e., the correct response and all the distractors described in the Section 3.6). The Raven-like matrices are generated according to either visuospatial or logic rules, which can be concatenated with the directional logics presented in Section 2.2 (i.e., vertical, horizontal, and diagonal). Finally, it is possible to print the generated matrices and the set of distractors either as separate images (i.e., each cell of the matrix and each distractor is printed separately) or as a single figure with the distractors. Stimuli generated with the matRiks package have been implemented on an online platform for evaluating fluid intelligence and planning skills in both general and clinical populations (i.e., PsycAssist; de Chiusole et al., 2024).

### 3.1 Installation

The matRiks package is available on CRAN and can be installed as:


**install.packages**(“matRiks”)


The code vignette(package = “matRiks") allows for obtaining the list of all the vignettes included in the package, which illustrate the default figures included in the package and a basic example of its application. Each vignette can be accessed via vignette("vignette-name," package="matRiks"). For instance, the code vignette("generate_matriks," package ="matRiks") opens the vignette that contains the instruction on how to generate an RMarkdown file where both the matrix and its associated response options are plotted together.

### 3.2 Definition of figures

The matRiks package contains several default figures that can be used for both the generation of the matrices and of new figures. The figures are based on the functions provided by the package DescTools (Signorell, 2025). All figures have class figure and they are defined as functions, such that the syntax figure_name() must be used to draw them and visualize their structures. The arguments that can be modified inside the parentheses may vary from figure to figure and allow us to change different features. The default features of the figures are stored in a list of length 15. The features of the figures are defined inside the list as follows:

- shape: character, the name of the figure
- size.x: numeric, the length of the semi-major axis of the ellipse within which the figure is inscribed (see the documentation of the DescTools for further details)
- size.y: numeric, the length of the semi-minor axis of the ellipse within which the figure is inscribed (see the documentation of the DescTools for further details)
- theta.1: numeric, radians of the rotation of the circle
- theta.2: numeric, radians of the rotation of the circle
- rotation: numeric, radians of the rotation of the ellipse within which the figure is inscribed
- pos.x: numeric, the position on the *x*-axis
- pos.y: numeric, the position on the *y*-axis
- lty: integer, the line type of the margins of the figure
- lwd: integer, the width of the margins of the figure
- num: numeric, equals 1 if the figure is drawn inscribed into an ellipse, and equals 2 if the figure is drawn as an arc of a circle
- nv: integer, the number of vertices of the figures (might vary from 2 – lines – to 100 – circle and ellipse –)
- shade: character, the filling of a figure (can also be NA –empty figure–)
- visible: integer, the visibility of the figure
- tag: character, properties of the figure used for the definition of the distractors

The tag of each figure describes some generic characteristics of the figure, which are used for the generation of the distractors. For instance, some tags indicate whether the figure is a single figure (simple), composed of two figures (compose2), or four figures (compose4). Other tags deal with the possibility of changing the shading of the figure (fill) or whether the figure can be rotated (rotate). The tags associated with a figure can be inspected with code figure()$tag. For instance, the tags associated with a square are:


**square**()**$**tag



##[[1]]
## [1] "simple" "fill"   "d.ext"  "rotate" 


These tags indicate that the square is a single figure (simple), it can be filled with different shadings (fill), and it can rotate (rotate). The tag d.ext specifically refers to the possibility of the figure being used for the generation of the Difference distractor (see Section 3.6). The tags associated with the Malta cross are "compose4", "fill", and "d.int", indicating that the figure is composed of four different figures, and it can be filled with different shading, respectively. Moreover, the absence of the rotate tag indicates that this figure cannot rotate.

**Figure 1:**
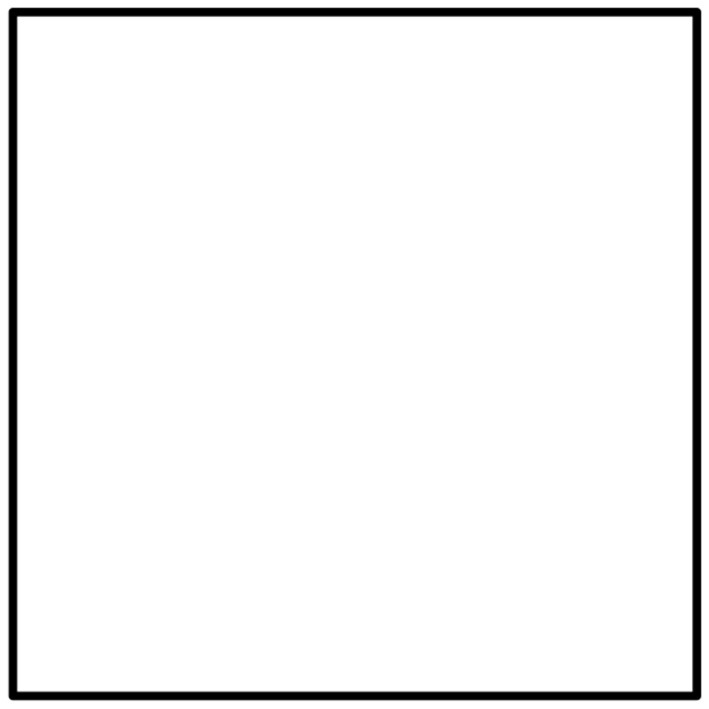
A simple square.

The draw() function is designed to print all the figures, as well as the matrices and the response list. To print a figure, the only argument needed inside the draw function is the name of the figure. For instance, a simple square (Figure 1) can be printed with the command line:


**draw**(**square**())


The figures in matRiks are summarized in different categories (e.g., closed figures, black figures, lines), for each of which a vignette lists all the figures. The categories of the figures available in matRiks, along with their associated vignettes, are illustrated in Table 2.

**Table 2:** List of figures and related vignettes.

**Figure category**	**Example**	**Figure category**	**Example**	**Figure category**	**Example**
Black Figures	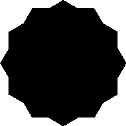	Flowers figures	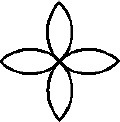	Other figures	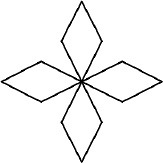
Circle sections	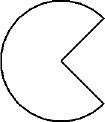	Eight-shaped figures	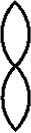		
Closed figures	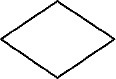	Lines	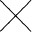		

### 3.3 Concatenation of figures

In addition to the pre-existing figures, the matRiks package allows for the generation of new figures by concatenating the existing ones. The cof() (**C**oncatenation **O**f **F**igures) function is designed for this aim. The arguments of the cof() function are the names of the default figures presented in the previous section.

**Figure 2:**
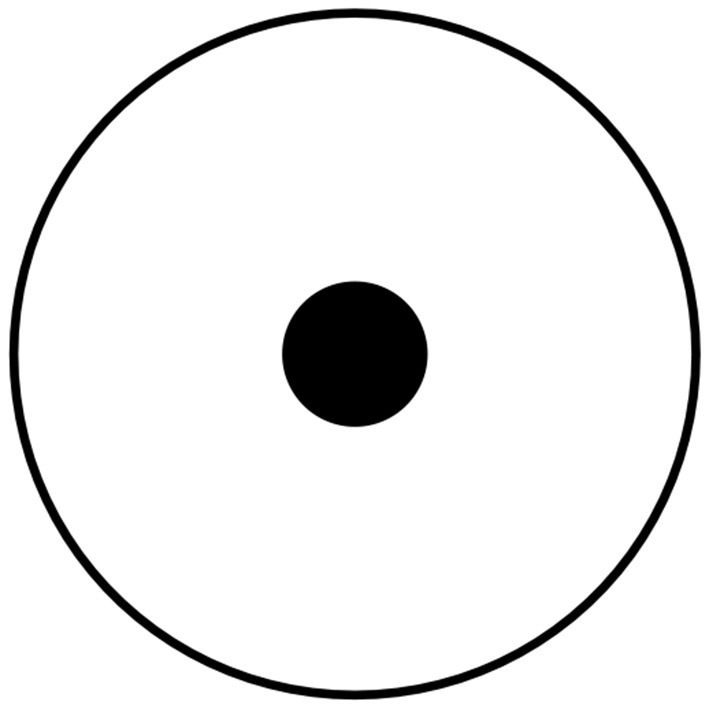
Example of concatenation of circle and dot to obtain an eye-like figure.

For instance, the figure in Figure 2 is obtained by concatenating a circle() and a dot().


# create the new figure “eye” by concatenating
the circle and the dot
eye <- cof(circle(), dot())
draw(eye)


The resulting figure eye is a named list of lists of class figure. However, the two original figures are still available and can be considered separate figures, and, as such, the object eye is considered a concatenation of two figures.


eye$shape



  ## [1] "circle" "dot" 
 


Additionally, function cof() has two optional arguments, namely single and name. If single = TRUE, the outcome of the concatenation is forced to be considered as a new single figure, while the second argument defines the new name for such a figure. The following code recreates the figure in Figure 2; however, it forces the new figure to be a single figure named “eye”.


s_eye <- cof(circle(),dot(), single = TRUE,
name = “eye”)
s_eye$shape



 
  ## [1] "eye" 
 


Although this difference is trivial from a graphical standpoint (the single figure and the concatenation of two distinct figures appear exactly the same), it is relevant for the application of some of the rules that require a minimum number of figures for their application (e.g., inferential rules, see Section 3.4).

### 3.4 Available rules and matrix generation

The function mat_apply(Sq1, hrules= "identity," vrules = "identity," mat.type = 9) is the main function for the generation of matrices based on rules. This function allows the generation of matrices of different dimensions, either four-cell or nine-cell. The dimension of the matrix is specified by the argument mat.type, such that mat.type = 4 results in four-cell matrices (left panel of Table 3) and mat.type = 9 results in nine-cell matrices (default, right panel of Table 3).

**Table 3:** Four-cell or 2 × 2 matrix (left panel) and nine-cell or 3 × 3 matrix (right panel).

Sq1	Sq2		Sq1	Sq2	Sq3
Sq3	Sq4		Sq4	Sq5	Sq6
			Sq7	Sq8	Sq9

The elements contained within a cell are denoted by “Sq”, followed by the cell number. The label Sq # in the left and right panels of Table 3 stands for “squares”. The Sq1 argument of the mat_apply() function defines the starting figure (i.e., the figure to be plotted in the first cell Sq1), which can also be a concatenation of figures. The arguments hrules and vrules allow for the definition of the directional logic with which the rule(s) are applied, such that the rules specified in hrules are manipulated horizontally, and those specified in vrules are applied vertically.

The application of the mat_apply() function results in a named list of class matriks that contains the characteristics of the matrix, such as the figures that are visible in each cell, the rule(s) applied horizontally and/or vertically, and the matrix type, either four-cell or nine-cell. The structure reported in the following example is based on a nine-cell matrix where the identity rule was applied both horizontally and vertically:


## [1] "Sq1" "Sq2" "Sq3" "Sq4" "Sq5" "Sq6"
## [7] "Sq7" "Sq8" "Sq9" "hrule" "vrule" "mat.type"


In a matrix, each cell is an element of the named list. Specifically, the cell entries are stored under names of the form Sq#, where # is the cell number (e.g., Sq1, Sq2, …). In the above example, nine elements (from Sq1 to Sq9) are listed corresponding to the nine cells as depicted in Table 3. In a four-cell matrix, four elements will be presented and named from Sq1 to Sq4. In addition to the Sq elements, the class matriks contains hrule, vrule, and mat.type. The first two are vectors containing the rule(s) applied horizontally and vertically, respectively. The last one is the dimension of the matrix.

The rules are methods that transform a feature of the figure to obtain a different figure or a figure with a modified feature. The matRiks package implements several rules, each of which may be classified differently. In particular, the original rule classification introduced in the literature, which is visuospatial and logical, has been modified in the matRiks package to focus on constructing matrices rather than solving them. Consequently, the rules are reclassified into incremental and permutational categories.

**Figure 3:**
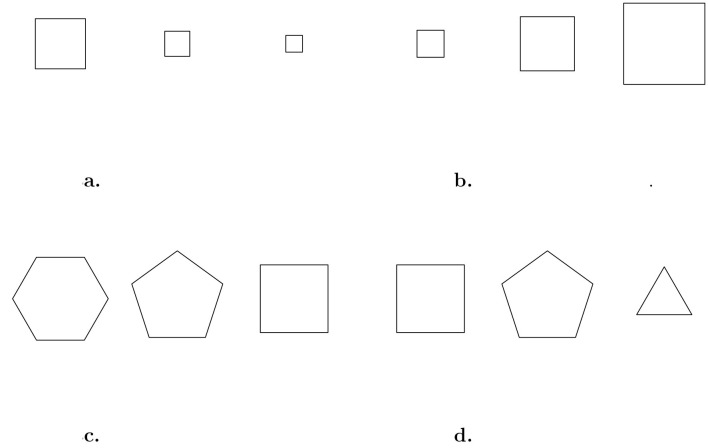
Examples of an incremental rule changes in size (top panels) with default **(a)** and reverse **(b)** application and of permuational rule changes in shape (bottom panels) with default **(c)** and reverse **(d)** application.

Figure 3a illustrates the application of the change in size rule on a square. The change-in-size rule is an incremental rule in which the size of the square decreases by a fixed quantity in each subsequent object according to a directional logic. Incremental rules apply a fixed increase (or decrease) on each cell of the matrix to obtain the features of the figure in the following cell, starting from the first cell. The order in which the fixed increase (decrease) is applied depends on the directional logic used for the generation of the matrix. This rule is incremental: the order of the squares in the cells is determined by a fixed variation in their sizes, from largest to smallest. If the positions of the squares across the cells are changed, then the definition of the rule is no longer satisfied, unless the squares are ordered from smallest to largest. In this case, the rule is still incremental but with a reverse application.

The operation underlying the permutational rules is the permutation of the figures (or their features) across the cells according to a directional logic. For instance, consider a matrix where a set of three figures (hexagon(), pentagon(), square()) are manipulated according to the shape rule following a horizontal directional logic (Figure 3c). By default, the figures are ordered according to the concatenation order of the set defined by the user with the cof() function. When the user changes the starting order, the rule will adapt accordingly.

All the rule functions are characterized by three arguments, namely fig, n, and rules. The fig argument specifies the initial figure to which the rule is applied. Some rules, such as size, can be applied to any type of figure, while others have specific constraints; for example, the shape rule requires the concatenation of three figures. Detailed definitions of the rules implemented in the package are provided in the “Definitions” column of Table 4.

**Table 4:** Types of rules implemented in the matRiks pacakge.

**Rule**	**Classification**	**Function**	**Definition**
Identity		Identity	Return the original figure without any transformation
AND	Logical	Logical	Considering the input cell fig, a concatenation of at least three figures is partitioned into three sets: A, B, and C. The rule transforms the figures such that each row or column in the matrix follows a specific sequence of patterns. In these patterns, the first cell displays figures {*A, C*}, the second cell displays {*A, B*}, and the third one exclusively displays figure A. The partitioning of the figures into sets A, B, and C is randomly determined with a random seed based on the n rows/columns of the matrix.
OR	Logical	Logical	Considering the input cell fig, a concatenation of at least three figures is partitioned into three sets: A, B, and C. The rule transforms the figures such that each row or column in the matrix follows a specific sequence of patterns. In these patterns, the first cell displays figures {*A, C*}, the second cell displays {*A, B*}, and the third one combines all of them in {*A, B, C*}. The partitioning of the figures into sets A, B, and C is randomly determined with a random seed based on the n rows/columns of the matrix.
XOR	Logical	Logical	Considering the input cell fig, a concatenation of at least three figures is partitioned into three sets: A, B, and C. The rule transforms the figures such that each row or column in the matrix follows a specific sequence pattern. In these patterns, the first elements display figures {*A, C*}, the second display {*A, B*}, and the third display {*B, C*}. The partitioning of the figures into sets A, B, and C is randomly determined with a random seed based on the n rows/columns of the matrix.
Line width	Permutational	Margin	Considering a figure or concatenation of figures, the lwd rule increases the width of the lines in the figure by a constant value n corresponding to the number of rows or columns in the matrix. Therefore, the width can have values 1, 2, or 3 of the default width argument of the R plot. Conversely, the reverse rule lwd.inv decreases the line width by the same quantity.
Line type	Permutational	Margin	Considering a figure or concatenation of figures, the lty rule changes the line type by manipulating the line type argument of the R plot. In the default order with the values of n from 1 to 3, the lines are dashed, dotted, and solid, respectively. Using the reverse rule, with the values of n from 1 to 3, lty.inv has the order dashed, solid, and dotted.
Rotate	Incremental	Rotate	Considering a figure or concatenation of figures and an angle θ, the rule rotates the figure around its center clockwise by an angle *nθ*, where *n* is the argument n of the function. The value of θ is equal to π divided by any number from 1 to 9 included in the rule argument of the function. For instance, rule=rotation.5 sets θ = π/5. By default, θ = π/4. The reverse rule rotation.inv rotates the figure anticlockwise.
Size	Incremental	Size	Considering a figure or concatenation of figures and a constant *k*, the rule size decreases the figure size proportionally to *nk*, where *n* is the argument n of the function. Specifically, the size.x and size.y arguments of the figure are divided by *nk*. The default value of *k* = 0.9. The reverse rule size.inv increases the figure arguments size.x and size.y by *nk* times, with a default value of *k* = 0.6.
Shape	Permutational	Shape	Considering a concatenation of three single figures denoted A, B, and C, the shape rule permutates which figure is visible in each cell of the matrix. The default order is figures A, B, and C from left to right in the row or from top to bottom in the column. The reverse rule shape.inv has the order C, B, and A.
Shade	Permutational	Shade	Considering a figure or concatenation of figures, the shade rule changes the color of the filling. The argument n of the function ranges from 1 to 3 and maps to white, gray, and black, respectively. The rule ignores any previous color present in the figure. For instance, when n = 1 and a figure has shd=black, the application of the rule transforms it into white. No reverse rule is available at the moment.
Multi shade	Permutational	Shade	Considering a concatenation of figures, the multi.shade rule changes the color of the filling of each figure separately. The rule works exactly as the normal shade, but a random color is assigned to each figure before the transformation. The random color is assigned with the function sample using seed(n).

The n argument specifies either the elements of the permutation or the increment value, depending on whether the rule is permutational or incremental. In mat_apply(), there is also a link between the cell number and the constant n, which ensures that the rule's directional logic is maintained.

Finally, a single function can apply different rules (see the ‘Function' column in Table 4). The rules argument allows us to specify which specific rule to apply. For example, the margin function manipulates line attributes of figures, with options to adjust line width margin(…, rules = "lwd") or line type margin(…, rules = "lty"). Another use of the rules argument is to reverse the default direction of rule application. The top panels of Figure 3 illustrate the application of incremental rules. For instance, size(…, rules = "size") decreases in size from right to left (see Figure 3a). By adding inv to the rules vector, the rule is applied in reverse order. Figure 3b shows the output of mat_apply(square(size.x=10), hrules = "size.inv") applied to a sequence of squares.

The reverse application of the rules can be done with permutational rules as well (bottom panels of Figure 3). By starting with the same set of figures as the one used to generate Figure 3c, the specification of the argument hrules = "shape.inv" would result in a reverse ordering of the figures across the cells (Figure 3d).

Logical rules AND, OR, and XOR are special case rules that cannot be applied in reverse.

It may appear trivial at this point that the transformation obtained from a rule becomes evident only through a comparison of figures. For instance, in Figure 3a, the change in size is noticeable only when two cells are considered together. The figure in the first cell is smaller than the one in the second cell, which is smaller than the one in the third, and so on. This straightforward concept underlies the creation of a matrix starting from a figure using mat_apply().

The procedure underlying mat_apply() consists of three serial steps. For the sake of simplicity, they will be illustrated for the nine-cell matrices only. The same reasoning applies to four-cell matrices. The first step is to generate a named list of all the cells from Sq1 to Sq9. Each cell contains the initial figure defined in Sq1, with no rules applied yet. The second step checks whether the arguments hrules or vrules contain a logical rule. If they do, an error occurs in the following cases: If the logical rule is combined with a visuospatial rule, if a logical rule is used to generate a four-cell matrix, or if two different logical rules are concurrently applied to the same figures with differing directional logic. Since applying logical rules requires a well-defined relationship between the cells of the matrix, only one logical rule can be applied at a time. If there is a logical rule and none of these error conditions are met, the procedure will generate the entire matrix in a single step.

If there are no logical rules, the procedure enters the third step. The rules are applied following a horizontal directional logic, meaning they are iterated across the elements within columns. Subsequently, the rules specified in vrules are applied using vertical directional logic, meaning they are iterated over the elements across rows.

Since the third and fourth steps are performed one after the other using the same rule, with both horizontal and vertical logic, the two transformations on the cells accumulate. For instance, consider the code mat_apply(square(), hrules="size," vrules="size"). At the end of the third step, the figure in Sq5 will be half the size of Sq1. At the fourth step, the size of Sq5 will be halved again, resulting in a figure that is a quarter of the initial Sq1.

It is worth noting that the horizontal and vertical combinations of the same rule define the diagonal directional logic. In particular, if the same rule is applied in reverse in one direction and in the forward direction in the other, the result is a TL-LR directional logic. On the other hand, if the same rule is direct (or reverse) with both horizontal and vertical directional logic, it will result in an LL-TR logic.

### 3.5 Concatenation of matrices

The com() (**C**oncatenation **O**f **M**atrices) function allows for combining and layering different matrices together to generate a multi-layer matrix.

**Figure 4:**
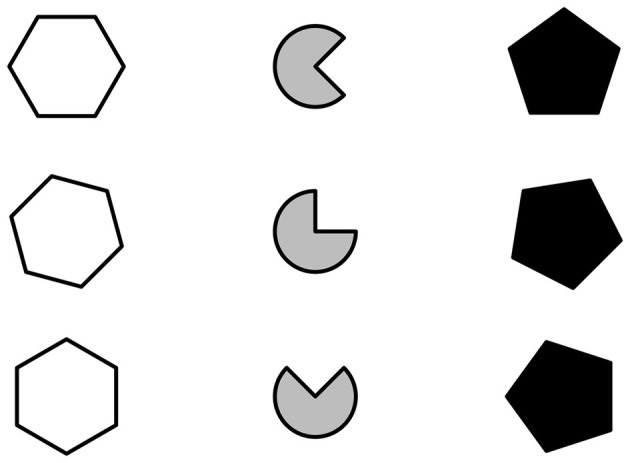
Single-layer matrix with two rules manipulated horizontally (Shape and filling) and one rule manipulated vertically (Orientation).

**Figure 5:**
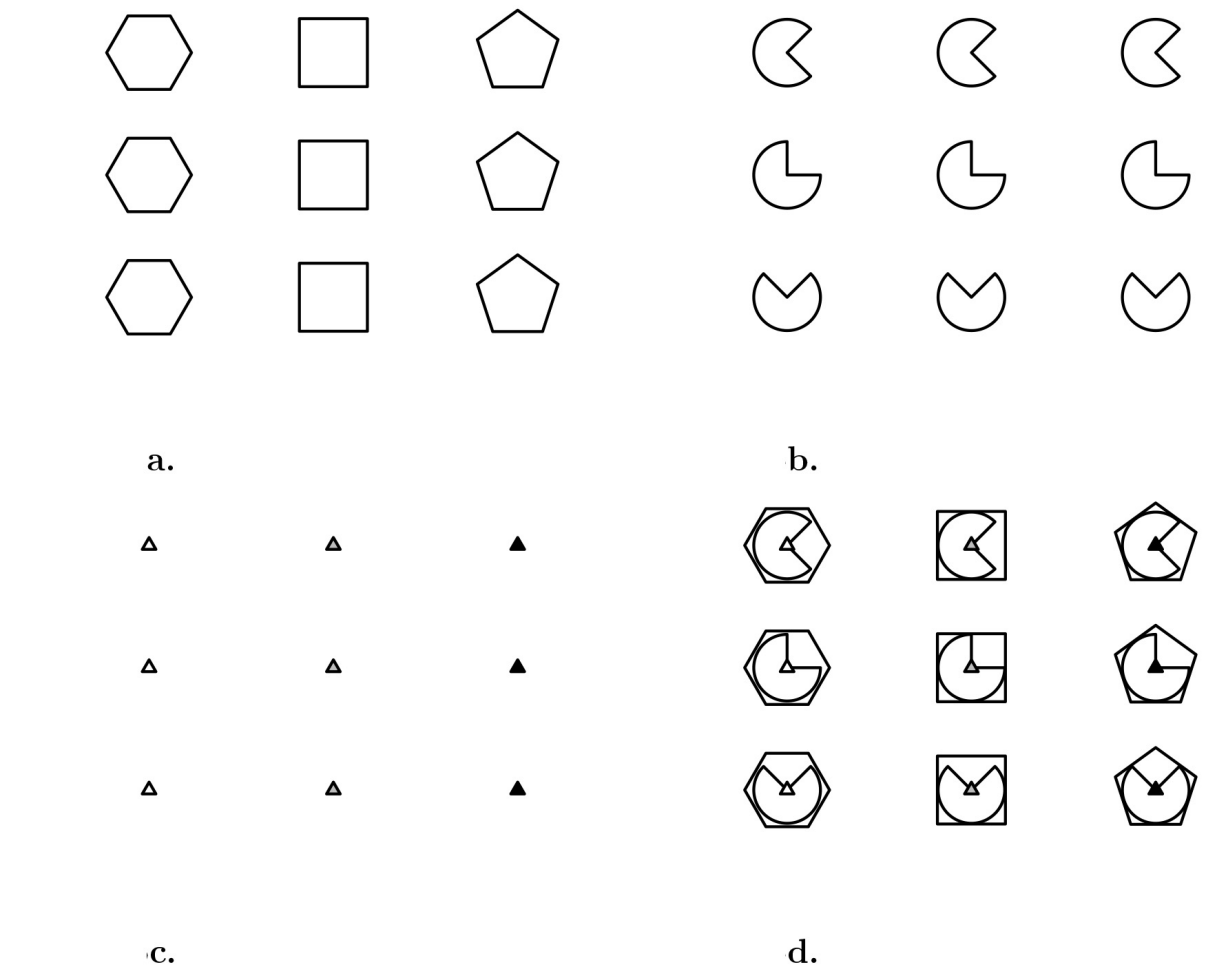
Multi-layer matrix **(d)** with two rules manipulated horizontally (shape and filling) and one rule manipulated vertically (orientation) obtained with the use of the com() function to concatenate the background layer **(a)**, the middle layer **(b)**, and the foreground layer **(c)**.

Figures 4, 5d depict a matrix created by manipulating the same set of rules. Specifically, two rules are manipulated horizontally (i.e., shade and shape) and one rule is manipulated vertically (i.e., rotate). As such, the figures in the cells and the shading change across columns, while the rotation of the figures changes across rows.

The matrix depicted in Figure 4 results from the manipulation of the set of rules to obtain a single-layer matrix, such that all rules are concurrently applied to the same set of figures (i.e., hexagon(), square(), pentagon()). The same set of rules is manipulated to obtain the matrix in Figure 5d. However, the three rules are manipulated to obtain three different single-layer matrices, one for each rule (Figures 5a–c), which are then concatenated together. Each of these single-layer matrices can be considered as a layer of the multi-layer matrix. The layering of the matrices moves from the background to the foreground. Layers are counted inwards *m =* 1, …, *M* (where *M* is the total number of matrices to concatenate), such that the background matrix is layer 1 and the foreground matrix is layer *M*. The matrix in Figure 5d is composed of *M =* 3 layers: (1) background layer obtained with the horizontal manipulation of the shape rule (Figure 5a), (2) middle layer obtained with the vertical manipulation of the rotate rule (Figure 5b, and (3) foreground layer obtained with the horizontal manipulation of the shade rule (Figure 5c).

The com() function concatenates the single-layer matrices to obtain the multi-layer one:


com(multi_a, multi_b, multi_c)


The matrices have to be concatenated hierarchically from the background layer to the foreground layer. The hierarchy among the layers is of utmost importance for defining the distractors, particularly for generating the IC ones. Generally, the manipulation of the correct response to obtain the IC distractors is applied to the figures in the foreground layer *M*. Further details on the definition and generation of the incomplete correlate distractors for multi-layer matrices are provided in Section 3.6.

### 3.6 Generation of the response list

The criteria for the classification of the error types presented in Section 2.3 were used for the formal definition and generation of the distractors in the matRiks package. The response options operator uses these criteria to generate a list, denoted as the response list, composed of 11 elements (ten distractors and the correct response), from which users can choose the most appropriate. Beyond the correct response, the response list contains: (i) three R distractors, (ii) one D distractor, (iii) two WP distractors, and (iv) four IC distractors, as illustrated in Table 5.

**Table 5:** Definition of the distractors implemented in the matRiks package for 3 × 3 and 2 × 2 matrices.

**Distractors**	**Layer**	**3 × 3 matrices**	**2 × 2 matrices**
R-Left	Both	Sq8	Sq3
R-Top	Both	Sq6	Sq2
R-Diag	Both	Sq5	Sq1
Wp-Copy	Single	Sq1 or Sq3	Sq1
WP-Matrix	Multi	Sq1 or Sq3 with the superimposition of another cell.	Sq3 or Sq2 with the superimposition of the rotation of WP-Copy
Difference	Multi	Sq1 or Sq3, Sq4, Sq7 with the superimposition of a figure which is not manipulated in the matrix.	Sq3 or Sq1 with the superimposition of a figure that is not manipulated in the matrix
IC-Inc	Single	Not possible	
	Multi	The foreground figure is removed	
	Multi	Logic matrices: The removed element is randomly selected	
IC-Neg	Single	Color inversion of the figure in the correct response	
	Multi	Color inversion of the most foreground figure of the correct response	
IC-Flip	Single	Reflection/rotation of the figure in the correct response	
	Multi	Reflection/Rotation of the foreground figure of the correct response	
IC-Scale	Single	Resize of the figure in the correct response	
	Multi	Only the most internal figure in the correct response is resized	

R, repetition distractors; WP, wrong principle distractors; IC, incomplete correlate distractors; IC-Inc, incomplete correlate-incomplete distractors; IC-Neg, incomplete correlate-negative distractors. The cell labels of the 2 × 2 and 3 × 3 matrices follow the nomenclature in the left and right panels of Table 3, respectively.

The response options operator constraints the distractors generation according to: (i) the type of matrix (i.e., single-layer matrix vs. multi-layer matrix), (ii) the rule(s) manipulated for the matrix generation, and (iii) the directional logic of the rule manipulation. To the best of our knowledge, there is no formal definition of the specific features of each distractor and their applicability given the above-mentioned constraints. As such, the formal definition of the distractors and of all the possible exceptions given the constraints imposed by the matrices generated via the matrix operator was needed for the implementation of the response options generator.

The response options operator (implemented in the response_list() function) runs through different steps. First, it checks the number of rules and the directional logic used to manipulate them, as well as whether the matrix is single-layer or multi-layer. This check is crucial for generating the IC distractors, but not for the other types (i.e., R, WP, and D), since they are generated by treating figures in a cell as a single figure. Conversely, since the IC distractors are a modification of a single feature of the correct response, there is a need to determine whether they can be generated in the first place and, if so, on which element of the correct response the change should be applied.

In single-layer matrices, the IC-Inc distractor cannot be generated, given that it requires the removal of an element of the figure, while all other IC distractors can be generated, unless a figure with specific tags is employed. For instance, S-shaped figures such as the miley() cannot change color, and thus the “shade” tag is absent from their tag list. When the response option generator does not find the shade tag among the tags of miley() to generate the IC-Neg distractor, it throws a warning, and the IC-Neg distractor is replaced by the correct response, canceled out by a thick black cross.

In multi-layer matrices, the IC distractors are generated by modifying the figure in the foreground matrix. Before applying the manipulation, the tags of the figure in the foreground matrix are checked to investigate whether all the IC distractors can be generated from that figure. If this is not the case, the response option generator moves toward the background of the multi-layer matrix, until it finds a figure whose tags satisfy the condition for creating all the IC distractors.

For the generation of WP and D distractors, the choice between using cell Sq1 and cell Sq3 depends solely on the number of rules and the directional logic used to manipulate them. Specifically, cell Sq1 is selected when the matrix is generated by manipulating a single rule with V, H, or TL-LR directional logic. If the manipulation of the single rule follows a LL-TR directional logic or two rules are manipulated with all possible directional logic, the Sq3 cell is chosen. This choice prevents WP-Copy distractors from being interpreted as IC distractors. In some instances, the R distractors in 3 × 3 matrices cannot be generated because they are equal to the correct response. For instance, if a matrix is generated with the vertical manipulation of a single rule, the R-Left distractor (Sq8) is equal to the correct response. In such instances, the distractor that equals the correct response is generated by the response options operator but is covered by a thick black cross, and the function generates a warning.

A similar procedure is applied for choosing which cell to use between Sq3 and Sq2 or between Sq3 and Sq1 for the generation of WP-Matrix and D distractors in 2 × 2 matrices. In both cases, the choice depends on whether at least one rule is applied both horizontally and vertically. If the same rule is applied both horizontally and vertically, Sq3 is chosen; otherwise, Sq2 and Sq1 are chosen.

**Figure 6:**
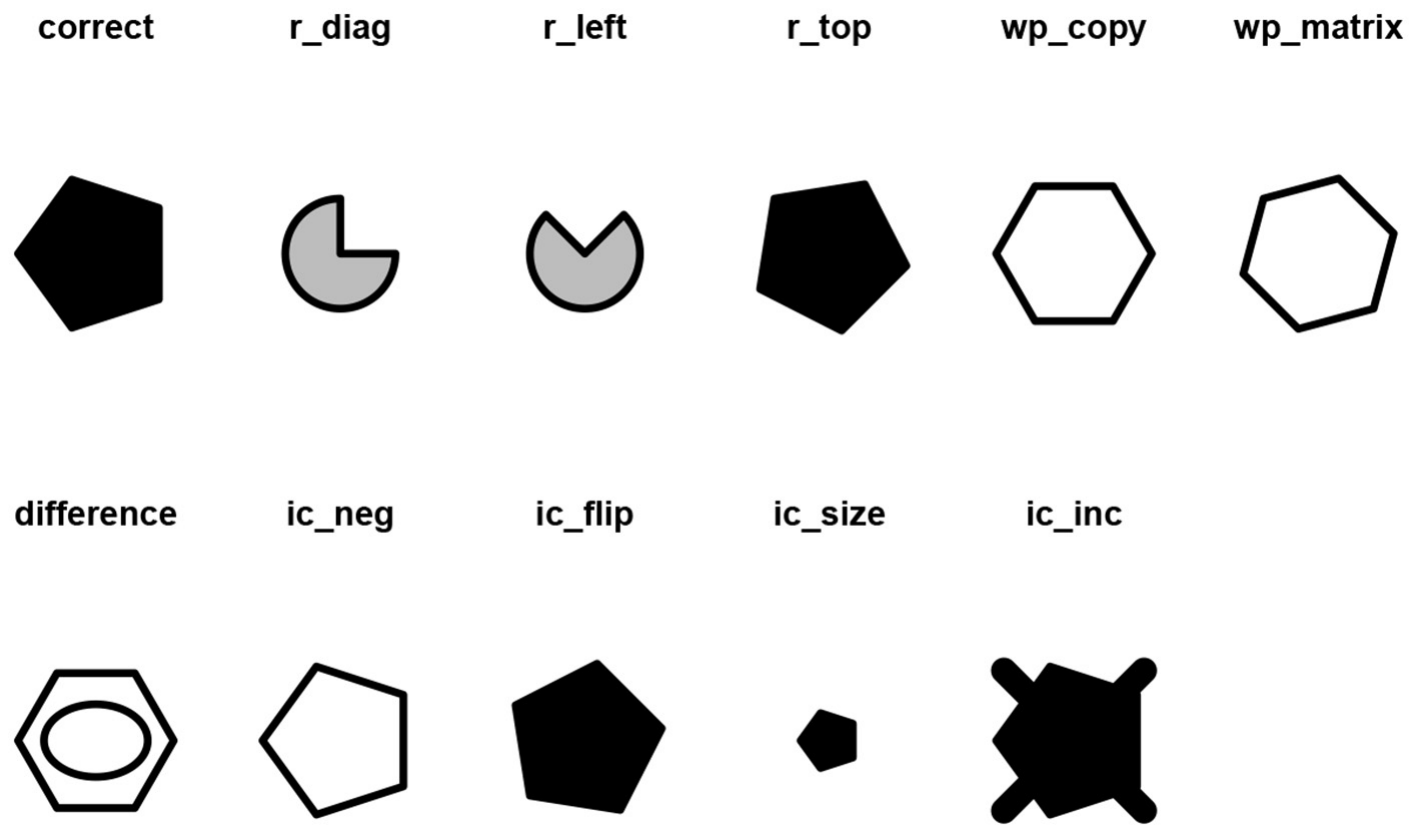
Distractors associated to the single-layer matrix in Figure 4.

The response options operator generates the response list (i.e., the correct response and all the distractors presented in the Table) through the response_list() function, which results in a list of figures that correspond to each of the distractors. For instance, the distractors of the matrix in Figure 4 can be obtained with Figure 6:


response_list(single_matrix)


The function response_list() results in a named list of length 11 containing all the response options:


## [1] "correct" "r_diag" "r_left" "r_top" "wp_copy"
## [6] "wp_matrix" "difference" "ic_neg" "ic_flip" "ic_size"
## [11] "ic_inc"


and it can be plotted with the draw() function:


 
  ## Warning in ic_inc.matriks(obj): IC-Inc
   cannot be obtained with a single figure
 


As can be noted, a warning has appeared regarding the IC-Inc distractor. As per Table 5, the distractor IC-Inc is defined as the correct response with a missing element. However, the single-layer matrix does not allow for the removal of any element; hence, the warning “IC-Inc cannot be obtained with a single figure” is thrown, and the IC-Inc distractor is replaced by the correct response over which a black thick cross is imposed.

**Figure 7:**
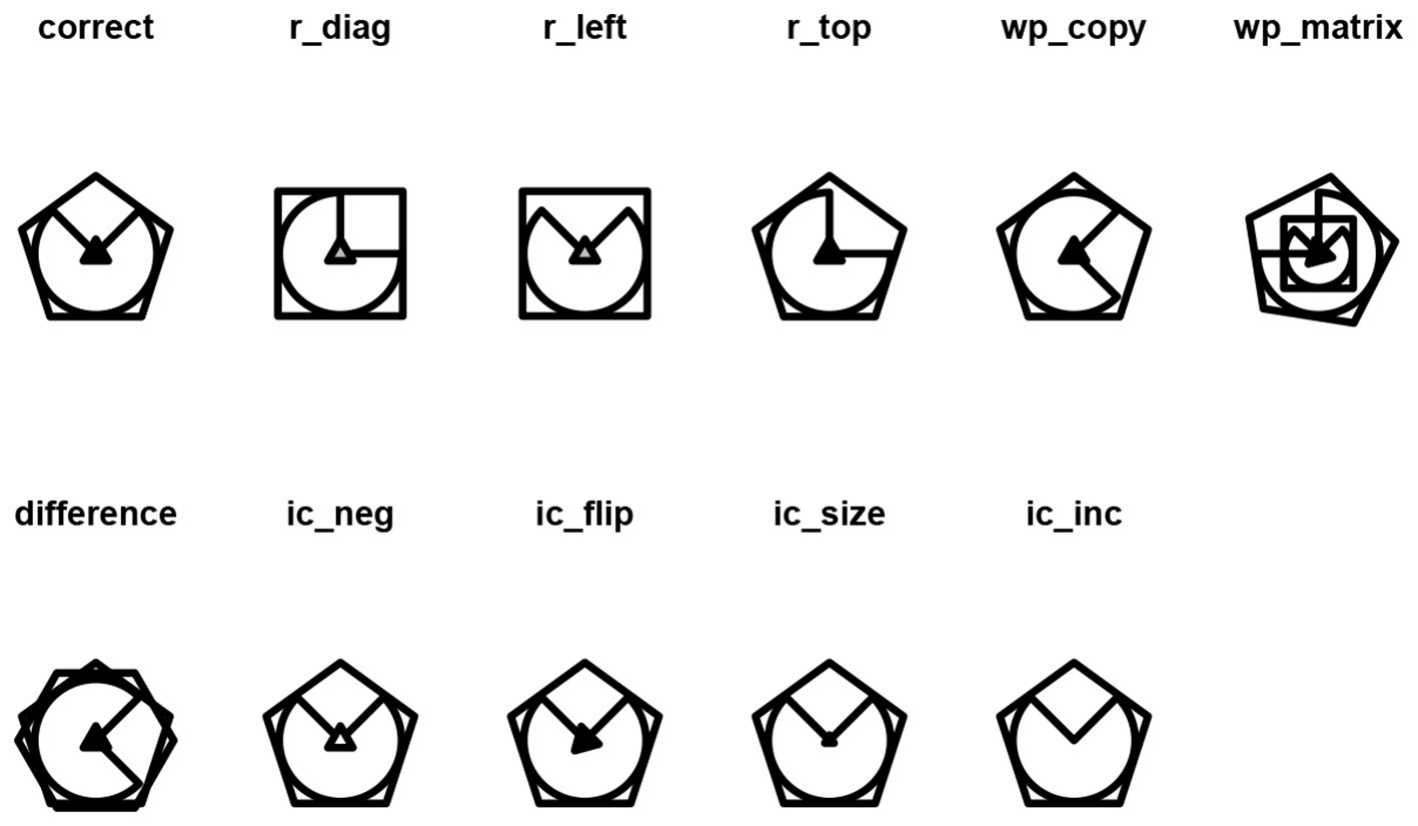
Distractors associated to the multi-layer matrix in Figure 5d.

Given that the matrix in Figure 5d is composed of three layers, it is also possible to obtain the IC-Inc distractor (Figure 7).

**Figure 8:**
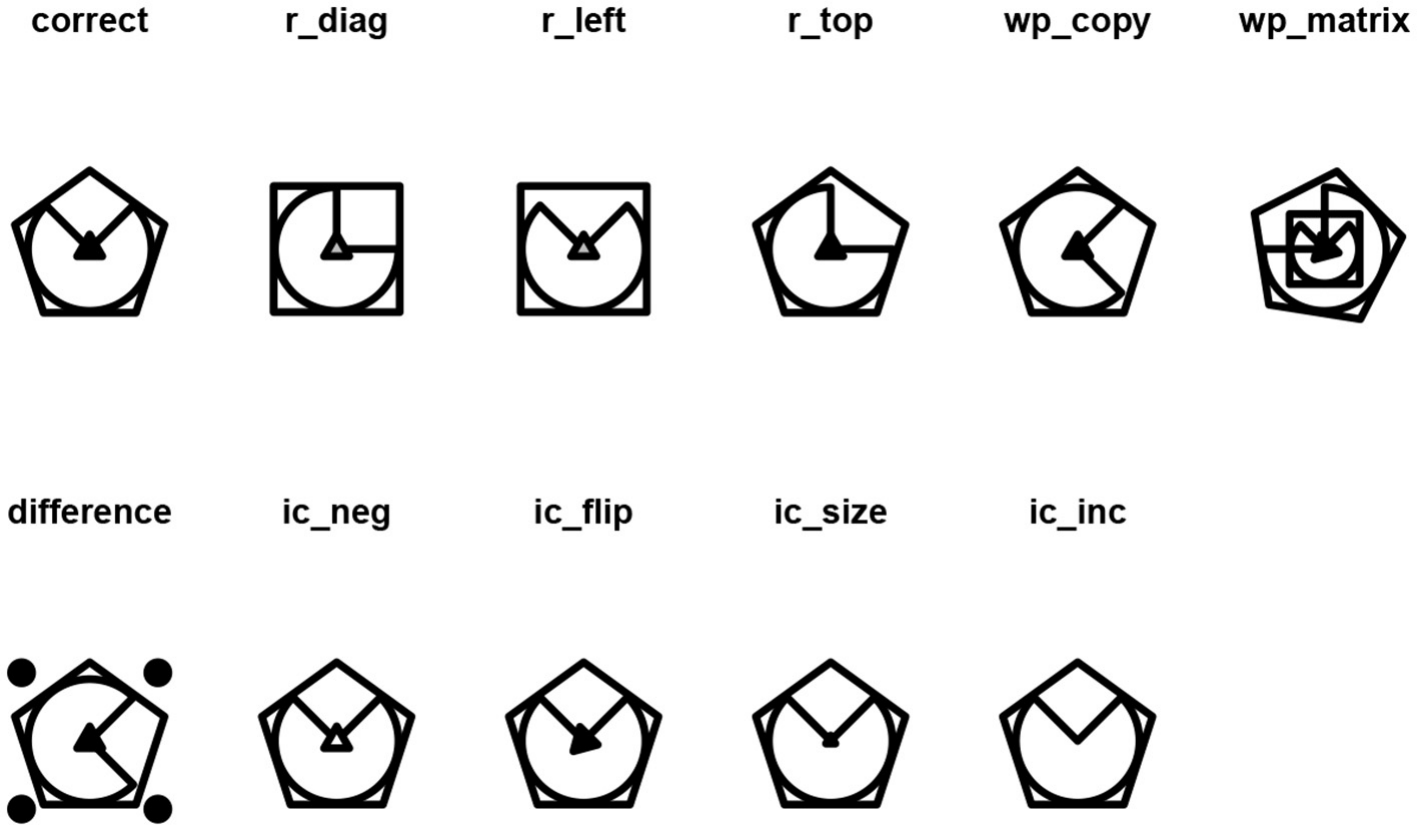
Modified distractors associated to the matrix in Figure 5d.

However, the difference distractor is not well defined. The user can change the random seed for the generation of this distractor with the argument seed(), such that another random figure is chosen among the available ones to generate the D distractor (Figure 8):


draw(response_list(multi_matrix, seed = 7),
main = TRUE)


By setting the argument main = TRUE, the names of the distractors as generated by the function response_list() are displayed along with the corresponding distractor. The user can change the original names by setting the argument labels to a character or numeric vector containing the desired labels. Further details are illustrated in the associated RMarkdown file (see Section 4).

As it stands now, the matRiks package does not allow manual customization of the generated distractors, although future versions will include this feature. Nonetheless, an example of how to change the shade of the generated distractors is illustrated in the associated R Markdown file (see Section 4).

## 4 Guided tutorial to stimulus generation

This section presents a complete example of generating a multi-layer 3 × 3 matrix using logical rules and its related response options. An R Markdown file with code to generate matrices of different dimensions with different rules is available in the Open Science Framework repository at https://osf.io/qsg7v/.

The first layer is generated by manipulating the AND logical rule according to an H-directional logic on a square composed of 4 lines:


logic1 <- mat_apply(square4(), hrules =
“AND”)


The second layer is generated by manipulating the OR logical rule according to a V-directional logic on a flower composed of 4 petals:


logic2 <- mat_apply(miley(), vrules = “OR”)


**Figure 9:**
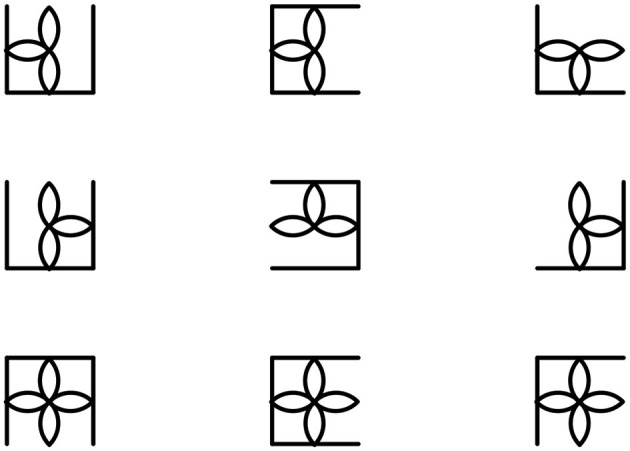
Multi-layer logic matrix.

The multi layer matrix (Figure 9) is created by concatenating the two single-layer matrices with the com() function:


logic <- com(logic1, logic2)
draw(logic)


**Figure 10:**
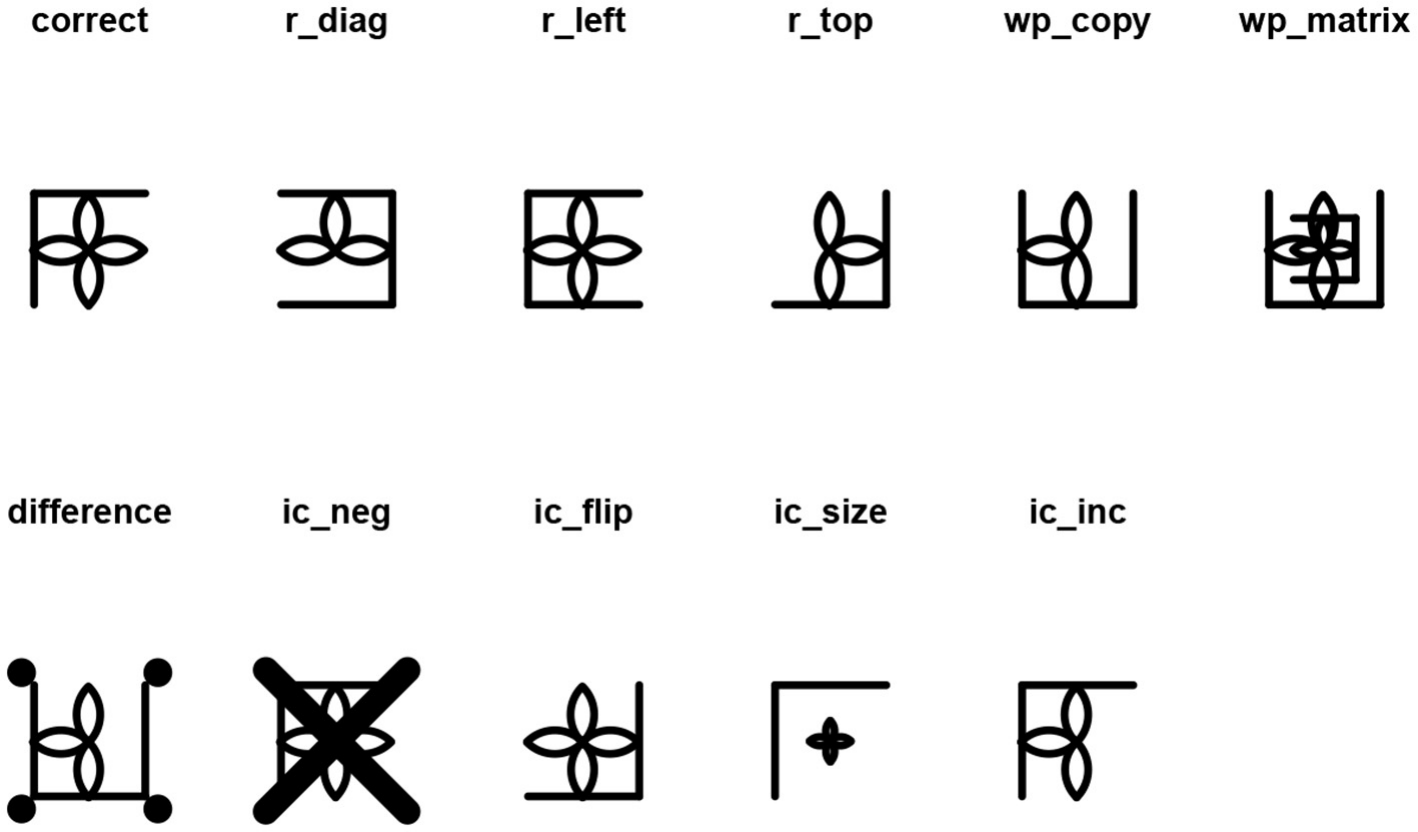
Distractors associated to the matrix in Figure 9.

The response options (Figure 10) associated with the multi-layer matrix in Figure 9 can be generated with the response_list() function:


responses <- response_list(logic)
draw(responses, main = TRUE)


Since the filling color of the miley cannot be changed, the IC-Neg distractor cannot be generated, the function throws a warning, and the ic_neg distractor is replaced by the correct response with the superimposition of a thick black cross. The difference distractor does not look good. The figure that is superimposed on the cell taken from the matrix can be changed by changing the seed in the response_list() function:


responses <- response_list(logic, seed = 12)
draw(responses, main = TRUE)


Assuming that a response list of length 8 (the correct response along with seven distractors) is associated with the multi-layer matrix, a character vector with the labels of the chosen distractors can be specified directly in the draw() function:


draw(responses,
distractors = c(“correct”, “r_diag”,
“r_left”, “wp_copy”, “wp_
matrix”, “difference”,
“ic_flip”, “ic_inc”))

## 5 Final remarks

This article illustrates the automatic generation of Raven-like stimuli with the matRiks package. The package has been developed to provide users with a flexible, open-source, and easy-to-use tool for generating Raven-like matrices according to different rules, encompassing both visuospatial and logical rules, along with their associated response lists.

The rules for generating the matrices and the distractors associated with each of them in the matRiks package derive from a vast body of literature on Raven's matrices and the error patterns observed on the CPM and the APM. As such, this package has the potential to generate stimuli that are equivalent to the standard Raven's stimuli in terms of rules and response options.

Moreover, this package provides a high degree of freedom in generating matrices of varying complexity. For instance, it allows the generation of stimuli that combine multiple rules by exploiting the ability to layer several matrices.

Although the functions implemented in the matRiks package are quite straightforward and easy to use, they require a basic knowledge of the R language. Moreover, advanced customizations, such as adding new figures that are not variations or concatenations of existing ones or modifying specific response options, still require an intermediate level of R proficiency. Future versions of the package will include easier ways to customize response options, add user-defined figures, and incorporate additional rules that are not currently included, such as “progression changes in numerosity”. Moreover, to facilitate use by users unfamiliar with coding, a graphical interface built with the shiny package (Chang et al., 2025) will be added in future versions of the package.

The package is regularly maintained, and new functions will be available in the future. As such, the users should refer to the official documentation of the package that is constantly updated.

Finally, the availability of tools that enable the generation of items for cognitive assessment, such as the matRiks package, supports the development of fully open tests, ensuring transparency of item construction and scoring procedures, methodological control, and independence from proprietary constraints.

## Data Availability

The original contributions presented in the study are included in the article/supplementary material, further inquiries can be directed to the corresponding author.
